# A conflict of interest: the evolutionary arms race between mammalian APOBEC3 and lentiviral Vif

**DOI:** 10.1186/s12977-017-0355-4

**Published:** 2017-05-08

**Authors:** Yusuke Nakano, Hirofumi Aso, Andrew Soper, Eri Yamada, Miyu Moriwaki, Guillermo Juarez-Fernandez, Yoshio Koyanagi, Kei Sato

**Affiliations:** 1 Laboratory of Systems Virology, Department of Biosystems Science, Institute for Frontier Life and Medical Sciences, Kyoto University, 53 Shogoinkawara-cho, Sakyo-ku, Kyoto, 6068507 Japan; 2 Faculty of Pharmaceutical Sciences, Kyoto University, Kyoto, Japan; 3 Graduate School of Biostudies, Kyoto University, Kyoto, Japan; 4 CREST, Japan Science and Technology Agency, Saitama, Japan

**Keywords:** Vif, APOBEC3, Lentivirus, Mammal, Evolutionary arms race

## Abstract

Apolipoprotein B mRNA editing enzyme catalytic polypeptide-like 3 (APOBEC3) proteins are mammalian-specific cellular deaminases and have a robust ability to restrain lentivirus replication. To antagonize APOBEC3-mediated antiviral action, lentiviruses have acquired viral infectivity factor (Vif) as an accessory gene. Mammalian APOBEC3 proteins inhibit lentiviral replication by enzymatically inserting G-to-A hypermutations in the viral genome, whereas lentiviral Vif proteins degrade host APOBEC3 via the ubiquitin/proteasome-dependent pathway. Recent investigations provide evidence that lentiviral *vif* genes evolved to combat mammalian APOBEC3 proteins. In corollary, mammalian *APOBEC3* genes are under Darwinian selective pressure to escape from antagonism by Vif. Based on these observations, it is widely accepted that lentiviral Vif and mammalian APOBEC3 have co-evolved and this concept is called an “evolutionary arms race.” This review provides a comprehensive summary of current knowledge with respect to the evolutionary dynamics occurring at this pivotal host-virus interface.

## Overview of lentiviruses and APOBEC3

### Classification of lentiviruses

**Fig. 1:**
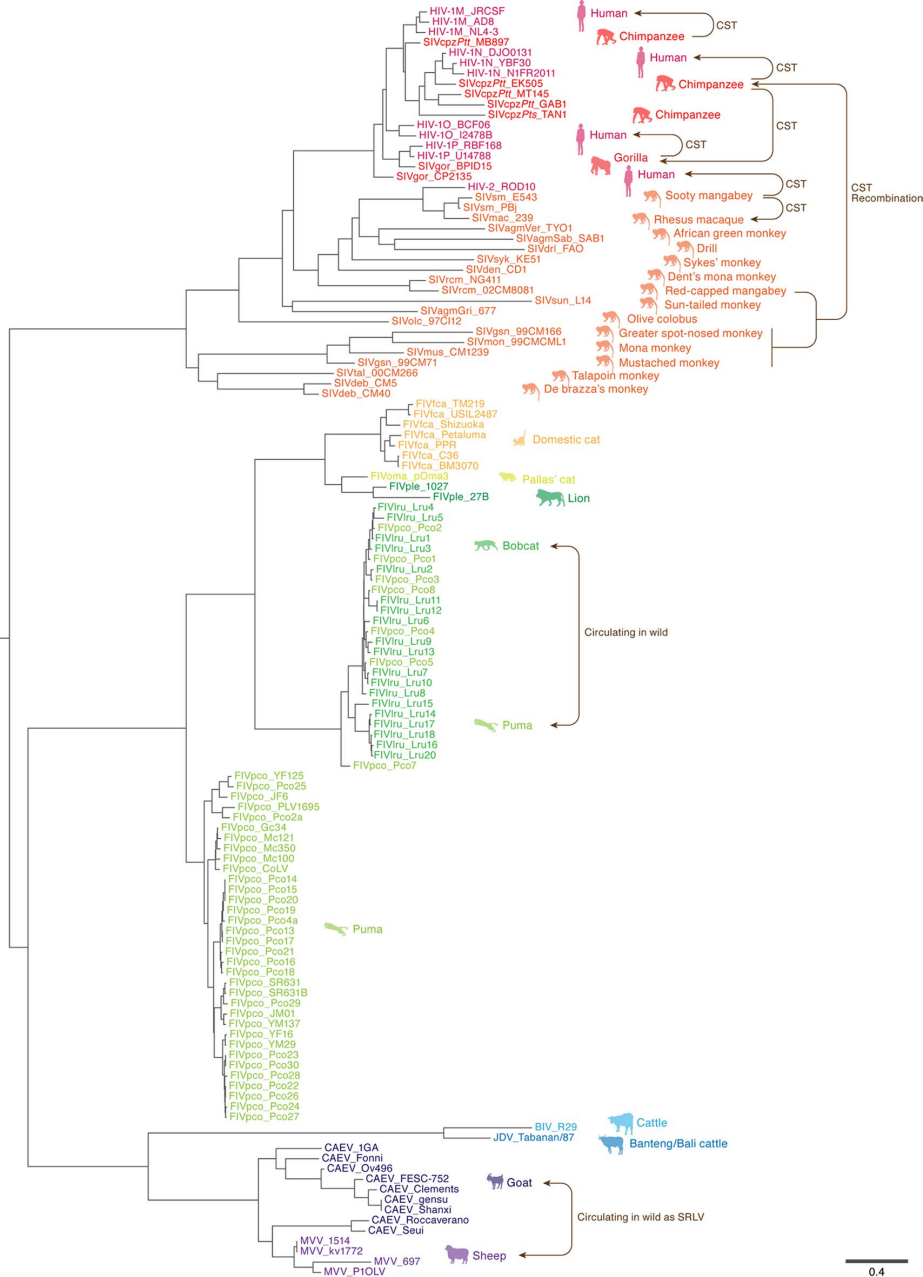
A phylogenetic tree of lentiviral Vif. The names of the viral families (e.g., SIVgor, FIVfca and CAEV) and their strains (shown after under the *bars*; e.g., CP2135, TM219 and Roccaverano) are labeled on the tips. The hosts of the respective viruses are represented on the right of each branch with an illustration. The estimated CSTs and a recombination of PLVs are indicated with *arrows*. The circulations of FIVlru/FIVpco (in bobcats and puma) and CAEV/MVV (in goat and sheep as SRLV) in the wild are indicated with the *double arrows*. The *scale bar* indicates 0.4 amino acid substitutions per site

Lentiviruses belong to the family *Retroviridae* and cause a variety of disorders in several species of mammals [1, 2]. In principle, exogenous lentiviruses are classified into five categories based on the host species: (1) primate lentiviruses (PLVs) in primates, (2) feline immunodeficiency viruses (FIVs) in felids, (3) bovine immunodeficiency virus (BIV) and Jembrana disease virus (JDV) in bovids, (4) Maedi–Visna virus (MVV) and caprine arthritis encephalitis virus (CAEV) in ruminants, and (5) Equine infectious anemia virus (EIAV) in horses (Fig. 1) [1, 2]. Human immunodeficiency virus type 1 (HIV-1) and type 2 (HIV-2) are PLVs and are known as the causative agents of acquired immunodeficiency syndrome (AIDS) in humans (*Homo sapiens*) (Fig. 1) (http://www.unaids.org/globalreport/). In addition, simian immunodeficiency viruses (SIVs) infect non-human primates including chimpanzees (*Pan troglodytes*), gorillas (*Gorilla gorilla*) and more than 40 species of Old World monkeys (OWMs) (Fig. 1).

### Characteristics of *APOBEC3* genes

Apolipoprotein B mRNA editing enzyme catalytic polypeptide-like 3 (APOBEC3; A3) proteins are cellular cytidine deaminases and are specifically found in mammals but not in other vertebrates [3, 4]. The A3 proteins of mammals, particularly those of primates, are considered to be cell-intrinsic immune factors that combat viruses, including lentiviruses and retrotransposons. To limit the replication of lentiviruses, the A3 proteins expressed in virus-producing cells are packaged into virions released from the cell. Then, A3 proteins are brought into neighboring cells and halt viral replication via enzymatic hypermutation of the viral genome and by blocking reverse transcription directly (for more detail, see references [5, 6]. As a result, lentiviral virions produced in the presence of A3 proteins are dramatically less infectious.

**Fig. 2:**
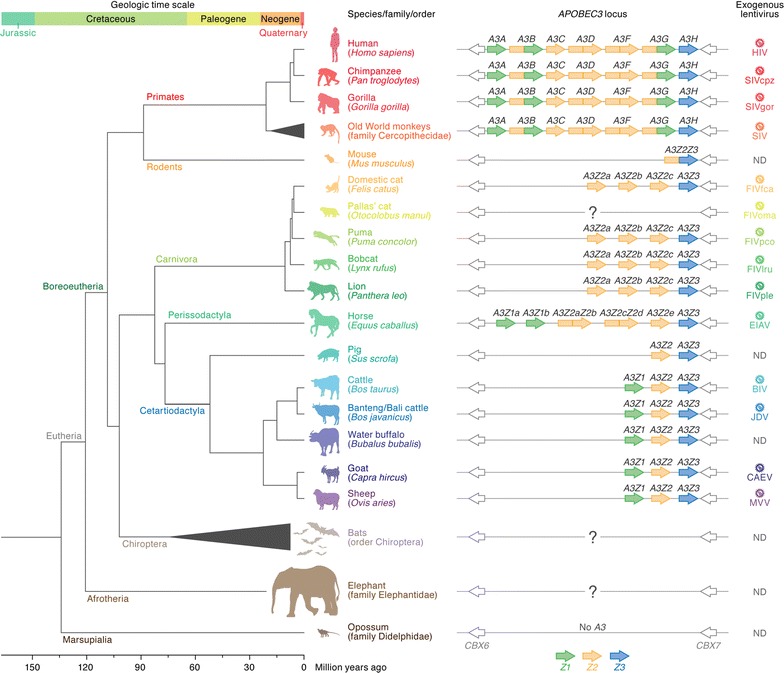
Evolution of mammals and their *A3* genes. *Left* A phylogenetic tree of mammals associated with lentiviruses with the geologic time (*top*) and age (*bottom*) scales is shown [14, 125]. In the phylogenetic tree, a subclass (Eutheria), a clade (Boreoeutheria), a superorder (Afrotheria), and some major orders (Primates, Rodents, Carnivora, Perissodactyla, Cetartiodactyla, Chiroptera, and Marsupialia) are labeled on each branch. Representative animals are illustrated with their common names and scientific names (in *parentheses*). *Middle* Schematics of the *A3* genes in each mammal are shown. The *A3* genes encoded in the *CBX6*-*CBX7* loci of respective mammals are summarized. The name of each *A3* gene is indicated on each open reading frame. *Z1, Z2* and *Z3* domains, which are classified by amino acid sequence, are shown in *green*, *yellow*, and *blue*, respectively [4]. Note that *A3* genes are not encoded in the genome of opossum [115]. The “?” indicates that the number and composition of *A3* genes in these mammals are currently unknown. *Right* The exogenous lentivirus in each mammal is shown. *ND* not detected (yet)

The mammalian *A3* genes are duplicated in a chromosomal locus flanked by *CBX6* and *CBX7* [4, 5, 7]. As summarized in Fig. 2, the number of *A3* genes and the history of the *A3* duplication process are different in each mammalian lineage (for more detail, see references [4, 5, 7, 8]. For instance, primates, including humans (*Homo sapiens*), have seven *A3* genes, while rodents including mice (*Mus musculus*) have only one ortholog. Horse (*Equus caballus*), pig (*Sus scrofa*), and bovids encode six, two and three *A3* genes, respectively (Fig. 2) [7, 8].

Based on the sequence homology of zinc-coordinating (Z) catalytic domains, mammalian A3 proteins are classified into three subsets: Z1, Z2 or Z3. Each A3 protein is composed of single or double Z domains [4]. For instance, human A3A, A3C and A3H encode single Z1, Z2 and Z3 domain proteins, respectively, whereas human A3B, A3D, A3F and A3G encode double Z domain proteins (Fig. 2) [4]. Previous molecular phylogenetic studies have indicated that the mammalian *A3* genes are evolving under positive selection [9, 10] and the gene duplications themselves likely result from selection pressures imposed by virus infections [11].

### An evolutionary arms race between mammals and lentiviruses

In the field of virology, revealing the co-evolutionary relationship between viruses and their hosts is intriguing and is crucial to understanding how viruses can impact the evolution of their hosts and vice versa. As summarized in Fig. 1, a hallmark of lentivirus ecology is the emergence of new lineages via cross-species transmission (CST) events. Additionally, viral recombination between lentivirus strains occurs frequently. As a result, reconstructing the dynamic relationship between lentiviruses and their hosts is complex.

To gain a better understanding of the evolutionary conflict between lentiviruses and their host species, cell-based virological experiments with a focus on the functional relationship between viral and host proteins have recently been conducted in combination with a molecular phylogenetic approach. This strategy stems from the concept known as the “Red Queen hypothesis [12]”, which proposes that games of cat-and-mouse occur between viral and host proteins as they engage with one another over time [13–15]. Based on this concept, various experiments have been conducted using mammalian A3 proteins and a lentiviral protein, viral infectivity factor (Vif).

### Vif-A3 interplay in terms of the CST events and the evolutionary arms race of lentiviruses and mammals

**Fig. 3:**
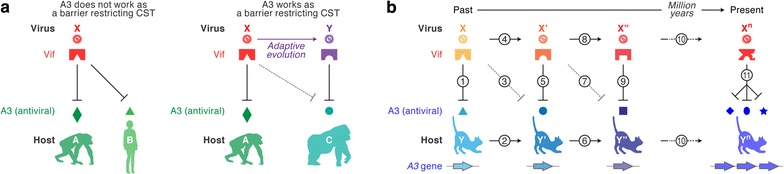
CST and the evolutionary arms race between viruses and hosts. **a** A3 as a barrier restricting CST. The lentivirus X infects the host A, and the Vif protein of virus X antagonizes the anti-viral A3 protein of host A. **a** *Left* When the Vif of virus X antagonizes not only anti-viral A3 of host A but also that of host B, a candidate for the new host, virus X can be successfully transferred from host A to host B without anti-viral restriction mediated by A3 of host B. **a** *Right* On the other hand, if the Vif protein of virus X is unable to antagonize anti-viral A3 of host C, another candidate for the new host, the anti-viral A3 of host C plays a role in restricting the CST of virus X from host A to host C. For a successful CST, the virus X evolves to adapt to host C and its Vif acquires the ability to antagonize host C’s anti-viral A3 (“Adaptive evolution” in this panel). As a result, a nascent species virus, the virus Y, has emerged and infects host C. **b** The concept of an evolutionary arms race between lentiviruses (Vif) and hosts (A3). In the past, an ancestral host (the host Y) was infected with an ancestral pathogenic virus (the virus X) and an anti-viral A3 protein of host Y was antagonized by the Vif protein of virus X (*1*). To escape from the pathogenic infection of virus X, the host A3 acquires certain mutations to be resistant to the degradation mediated by the virus X Vif, resulting in the emergence of a novel host, the host Y′ (*2*). Although the anti-viral A3 of the host Y′ is resistant to the virus X Vif (*3*), the virus X Vif acquires mutations to adapt to the host Y′ (*4*). Then, a novel virus, the virus X′, emerges and its Vif is able to antagonize an anti-viral A3 of the host Y′ (*5*). Subsequently, similar to the process of (*1*) to (*5*), the selective pressure by the virus X′ produces the host Y″ of which A3 is resistant to the virus X′ Vif (*6* and *7*), while the virus X′ evolves to antagonize the host Y″ A3 and becomes the virus X″ (*8* and *9*). Such an “arms race” between lentiviruses and hosts has evolutionarily occurred over a long period of time (*10*). It is speculated that this process might trigger the duplication of *A3* genes in mammals. After *n*-times arms races, in the present, the host Y^n^ encodes multiple anti-viral A3 proteins, while the Vif of the virus X^n^ antagonizes them (*11*)

Vif is an accessory protein encoded by all lentiviruses with the exception of EIAV, a lentivirus found in horses [1, 2]. As described above, the replication of lentiviruses lacking a functional Vif protein is robustly impaired by certain A3 proteins in the host. In contrast, lentiviral Vif degrades the A3 proteins expressed in virus-producing cells in an ubiquitin/proteasome-dependent manner to antagonize the A3-mediated antiviral action [5, 6, 16]. As described in Fig. 3, knowledge regarding the functional interaction between Vif and A3 can inform the quest to elucidate the principle of CST events and the subsequent evolutionary arms race between lentiviruses and their hosts. In the following sections, we describe our current understanding of the evolutionary relationships between lentiviruses and their hosts, which has been informed by functional interactions between lentiviral Vif and host A3 proteins.

## A3 antagonism by lentiviral Vif proteins

### Human A3 versus HIV

In 1983 and 1986, HIV-1 [17, 18] and HIV-2 [19] were isolated as the causative agents of AIDS, respectively. Both viruses likely emerged following independent CST events (Fig. 1), and molecular phylogenetic investigations have indicated that the origin of HIV-1 is SIVcpz (an SIV infecting chimpanzee [*Pan troglodytes*]) [20] or SIVgor (an SIV infecting gorilla [*Gorilla gorilla*]) [21], while the origin of HIV-2 is SIVsm (an SIV infecting sooty mangabey [*Cercocebus atys*]) [22]. Molecular clock analyses have estimated that the CST of SIVcpz (i.e., the birth of HIV-1) occurred ~100 years ago (around A.D. 1908–1933) [23], while the CST of SIVsm took place ~200 years ago (around A.D. 1729–1875) [24]. Moreover, HIV-1 is classified into 4 groups, M (main or major), N (new or non-M-non-O), O (outlier), and P, and each group has emerged following an individual CST event [20, 21, 25, 26] (Fig. 1). It should be noted that the virus causing the worldwide pandemic is HIV-1 group M [27]. The details of HIV pathogenesis and the clinical information through the study of infected individuals are well summarized elsewhere (e.g., see the following references: [2, 28, 29]).

In 2002, Sheehy et al. [30] identified *A3G* (this gene was originally designated *CEM15*) as a novel restriction factor against HIV-1 replication. Importantly, the A3G-mediated antiviral action is antagonized by HIV-1 Vif [30]. This report initiated research interest in Vif-A3 interactions and the impact of this interaction on HIV replication and pathogenesis (reviewed in references [5, 6, 16]). Now it is known that human (*Homo sapiens*) encodes seven *A3* genes on chromosome 22 (Fig. 2). Specifically, endogenous A3D, A3F, and A3G expressed in human CD4^+^ T lymphocytes, a major target cell type for HIV infection, display robust anti-HIV activity in cell culture-based experiments (in vitro) and humanized mice (in vivo) [5, 6, 16, 31–34]. Although other human A3s, such as A3A and A3B, have exhibited anti-HIV-1 activity in overexpression experiments [35, 36], it seems controversial whether endogenously expressed A3A and A3B can elicit anti-HIV effects [37, 38]. Human A3C exhibits weak anti-HIV-1 activity [39, 40], but a recent paper has demonstrated that a single nucleotide polymorphism in human A3C (S188I substitution) results in enhanced HIV-1 inhibition in cell-based overexpression experiments [41].

Intriguingly, OhAinle et al. [42] have reported that *A3H* is polymorphic in human populations. A follow-up study showed that there are at least seven haplotypes and that three of them, called haplotype II, V, and VII, produce stably expressed proteins and exhibit anti-HIV-1 activity [43]. In contrast, the other four haplotypes (I, III, IV, and VI) do not exhibit stable protein expression [43]. Since chimpanzee *A3H* appears to be monomorphic, and the protein encoded is stable [42], it appears that the human A3H protein has developed reduced stability since the divergence from chimpanzee. Furthermore, the frequency of each haplotype is different among human populations, with a higher frequency of stable *A3H* in the African-descendant population and a lower frequency in other populations such as European, American and Asian populations [42, 43].

Although the ability of HIV-1 Vif to antagonize antiviral human A3s including A3D, A3F, and A3G is highly conserved [44], the ability of Vif to degrade stable A3H is not; the Vif proteins of some HIV-1 isolates (e.g., strains LAI, 93RW037 and 93BR029) are capable of degrading stable A3H (e.g., haplotype II), while other isolates (e.g., strains NL4-3, 92TH026, AD.MDR01 and 93TH305) are not [45]. Mutagenesis experiments determined that the Vif residues at positions 39 and 48 are closely associated with the ability to counteract stable human A3H [35, 46, 47]. These observations suggest that the extent of anti-HIV-1 ability elicited by endogenous A3H varies among humans, and the gain and loss of the function of HIV-1 Vif to counteract stable A3H has repeatedly occurred during viral spread in vivo and human population.

### Non-human primate A3 versus SIV

The non-human primate hosts that are naturally infected with PLV in the wild can be divided into two groups: the OWMs in sub-Saharan Africa and the great apes, including chimpanzees (*Pan troglodytes*) and gorillas (*Gorilla gorilla*) (Fig. 1) [1]. Similar to humans, both OWMs and great apes encode seven *A3* genes in their genomes.

As summarized in Fig. 1, a variety of OWM species reside in Africa, and most of them harbor a species-specific SIV that is not overtly pathogenic [48–51] suggesting that SIVs have co-existed with their OWM hosts for a very long time [52]. In contrast, there is also evidence for more recent CST events that give rise to new lentivirus infections. Bailes et al. [26] have reported that SIVcpz emerged through the recombination of two SIV lineages: SIVrcm (an SIV infecting red-capped mangabeys [*Cercocebus torquatus*]) and SIVgsn/mon/mus (SIVs infecting greater spot-nosed monkeys [*Cercopithecus nictitans*], mona monkeys [*Cercopithecus mona*] and mustached monkeys [*Cercopithecus cephus*]) (Fig. 1), and this recombination event is assumed to have occurred ~500 years ago (around A.D. 1266–1685) [24]. SIVgor appears to have emerged through the CST of SIVcpz [21]. In sharp contrast to SIV infection in OWMs in Africa, exogenous PLVs have not been found in the New World.

As briefly described in the introduction, Vif-A3 interactions can provide clues to elucidate virus-host (i.e., lentivirus-mammal) evolutionary relationships and history. Here, we introduce three major examples suggesting that host A3 proteins may represent a barrier to inhibit CST: (1) rhesus macaque (*Macaca mulatta*) A3G is resistant to degradation mediated by HIV-1 Vif (strain NL4-3) [53, 54]; (2) rhesus macaque A3G is resistant to degradation mediated by SIVagm (an SIV infecting African green monkeys [*Chlorocebus aethiops*]; strain TAN-1) Vif [53]; and (3) gorilla A3G is resistant to degradation mediated by the Vif proteins of SIVcpz (e.g., strains LB715, MT145, EK505 and Gab2), the putative ancestor of SIVgor [25]. In contrast to example (3), SIVgor Vif is able to degrade and counteract gorilla A3G [25], suggesting that this virus has gained the ability to counteract gorilla A3G through adaptation to gorilla following CST from chimpanzee. Moreover, Compton and Emerman have recently demonstrated that the Vif proteins of SIVs naturally infecting OWMs, such as SIVagm, SIVmus, SIVsm, SIVdeb (an SIV infecting De brazza’s monkeys [*Cercopithecus neglectus*]) and SIVolc (an SIV infecting colobus monkeys [*Procolobus verus*]) (Fig. 1), counteract A3G proteins in a species-specific manner [55]. The species specificity of Vif action arises because many species encode point mutations in the region of A3G recognized by Vif [55]. These findings suggest that each SIV Vif has evolutionarily acquired the ability to overcome the restriction imposed by A3G of their respective hosts. Furthermore, three codons have been inserted into the *A3G* gene of the *Colobinae* primate subfamily, rendering it resistant to most SIV Vif proteins. The recurrent mutation of A3G at sites important for sensitivity to Vif suggests that Vif or Vif-like viral proteins must have applied selective pressure in ancestral primates. This implies that these ancient lentiviruses caused lethal pathogenesis and/or reproductive defects that drove evolutionary change in primate hosts. By dating the appearance of adaptive mutations in primate A3G, it has been inferred that SIV has been infecting non-human primates for at least 12 million years.

Similar to the case of human *A3H* (described above), *A3G* polymorphism has been observed in OWMs and is believed to affect viral transmission within and between species [55–58]. Furthermore, the antiviral potency can be divergent between different A3 proteins in the same host. For example, human A3F exhibits higher antiviral activity than human A3D, while chimpanzee A3F exhibits lower antiviral activity than chimpanzee A3D [59]. Altogether, the following issues make unraveling the functional interaction of PLV Vif and primate A3s and its consequence a challenge: the multiplicity of primate *A3* genes, the difference in anti-viral activity among *A3* genes and their hosts, inter- and intra-species heterogeneity of *A3* gene sequences, and divergence in PLV *vif* genes. Therefore, future investigations addressing the Vif-A3 interaction using experimental and comprehensive approaches through the techniques of molecular phylogenetic, evolutionary biology and mathematic modeling will be important to elucidate detail of the evolutionary arms race between PLVs and mammals.

### Carnivore A3 versus FIV

FIV was first isolated in 1987 from domestic cats (*Felis catus*) with chronic AIDS-like disorders [60]. Epidemiological studies by ELISA, immunoblotting, and/or PCR techniques have revealed that FIV is detectable in a broad range of wild animals in the family *Felidae* [61–63]. FIV has been detected in felids residing in the Old World, including lions (*Panthera leo*), cheetahs (*Acinonyx jubatus*) and leopards (*Panthera pardus*), and those in the New World, including pumas (*Puma concolor*), jaguars (*Panthera onca*), and ocelots (*Leopardus pardalis*) [61]. FIV appears to be distributed worldwide, since a felid species in Asia, Pallas’ cat (*Otocolobus manul*), has been shown to be positive for FIV by PCR [61, 64, 65]. Furthermore, one study has confirmed that a FIV-related virus is present in spotted hyenas (*Crocuta crocuta*), a related carnivore family belonging to *Hyaenidae* [61]. In contrast to the disorders seen in FIV-infected domestic cats, it appears that FIV infections in wild felids are relatively benign and apathogenic because of less divergence in the viral sequences and no obvious symptoms in the infected felids [66]. Therefore, it has been assumed that FIV has co-existed with felids for a long period of time, as in the case of SIV infection in OWMs (see above). The higher FIV pathogenicity in domestic cat is reminiscent of the observation of SIV infection in rhesus macaques (*Macaca mulatta*): SIVmac (an SIV infecting rhesus macaque [*Macaca mulatta*]) is highly pathogenic in this monkey and emerged from a CST of SIVsm from sooty mangabeys in 1985 (Fig. 1) [67]. Therefore, similar to the situation of SIVmac emergence, FIVfca might also have emerged from a relatively recent CST event from wild felids.

Because surveys designed to detect FIV have largely utilized serological methods (e.g., ELISA and immunoblot), FIV *vif* sequences are only available for the following viruses: FIVfca (an FIV infecting domestic cats [*Felis catus*]), FIVoma (an FIV infecting Pallas’ cats [*Otocolobus manul*]), FIVpco (an FIV infecting pumas [*Puma concolor*]), FIVlru (an FIV infecting bobcats [*Lynx rufus*]), and FIVple (an FIV infecting lions [*Panthera leo*]) (Fig. 1). Although each FIV has adapted to a specific host felid, recent studies have suggested that FIVlru and a subclass of FIVpco co-circulate in both puma and lynx in North America (Fig. 1) [68, 69].

Felids, including domestic cats (*Felis catus*), pumas (*Puma concolor*), bobcats (*Lynx rufus*) and lions (*Panthera leo*), encode multiple A3 proteins: three A3Z2 proteins (A32a, A32b and A32c; the orthologs of primate A3C) and a single A3Z3 (the ortholog of primate A3H) (Fig. 2) [70, 71]. In addition, a two-domain A3Z2Z3 hybrid protein is generated through alternative splicing [70, 71]. Similar to the relationship between primate A3s and PLVs (see above), previous studies revealed that the feline A3Z3 and A3Z2Z3 proteins potently suppress the infectivity of *vif*-deleted FIVfca [70–75] and that the Vif proteins of FIVfca (strains Petaluma, C36 and Shizuoka) counteract the antiviral action of domestic cat A3Z3 and A3Z2Z3 [71, 74].

Interestingly, de Castro et al. [76] have reported that there are at least seven *A3Z3* haplotypes in domestic cats (haplotypes I to VII) containing four nonsynonymous polymorphisms at codons 65, 68, 94, and 96, and a synonymous substitution at codon 100. A follow-up molecular phylogenetic analysis determined that codon 65 is under positive selection [74]. Intriguingly, cell-based experiments revealed that domestic cat A3Z3 haplotype V (A65I substitution compared to haplotype I) is resistant to degradation mediated by the FIVfca Vif proteins (strains Petaruma, C36 and Shizuoka) [74]. BEAST (Bayesian evolutionary analysis by sampling trees) analysis further implied that domestic cat A3Z3 haplotype V emerged approximately 60,000 years ago [74]. These observations strongly suggest that domestic cat A3Z3 haplotype V has been naturally selected to escape from an ancestral FIV and that an evolutionary arms race between FIVfca (or its ancestor) and domestic cats has taken place.

In addition to the interaction between FIVfca and domestic cat A3s, a previous paper has revealed that FIVfca (strain Petaluma) Vif is able to antagonize A3Z3 and A3Z2Z3 of other felids, including pumas, lions and tigers [71, 75]. In contrast, FIVpco (strain PLV-1695) Vif is unable to degrade domestic cat A3Z3 [75]. Most intriguingly, FIVpco (strain PLV-1695) Vif is incapable of counteracting A3Z3 of pumas, which are its natural host [75]. This is the first report demonstrating that a non-primate lentiviral Vif protein is unable to degrade an antiviral A3 protein of its natural host. Then, how is it possible that FIVpco Vif does not counteract A3 of its natural host? One possibility is that this may result in the elimination of this virus from circulation. However, a recent study by Lee et al. [77] have demonstrated an on-going cross-species circulation of FIVpco in puma and bobcat in North America. Additionally, as a higher viremia has been detected in the pumas infected with FIVpco [77], these observations argue against this possibility. Another possibility is that puma A3Z3 may provide only partial protection. Since the expression levels of endogenous *A3* genes in puma remain unassessed, it may be plausible to speculate that the anti-viral effect of endogenous puma A3Z3 is negligible. However, endogenous anti-viral A3 proteins are expressed in domestic cats and exhibit robust activity against FIVfca [78], Yoshikawa et al. [79] have recently demonstrated that the Vif proteins of a subtype of FIVfca (subtype B) lose their ability to antagonize domestic cat A3-mediated anti-viral effect despite the worldwide spread of this subtype. These findings imply that the Vif-A3 interaction is more complicated than expected, at least in the interplay between FIV Vif and feline A3. Therefore, future investigations should focus on elucidating the role of feline A3 in controlling feline lentiviruses and their cross-species circulation in the wild.

As described above, several FIV lineages have been isolated from various felids; however, the molecular interaction between their Vif proteins and the A3 proteins of their hosts remains unclear. Also, the importance of Vif antagonism of A3 proteins may depend on the host-virus context, and may suggest differences in lentivirus replication and pathogenesis in vivo. Therefore, investigation of the interplay of FIV Vif and the A3 proteins of *Felidae* will lead to a better understanding of an intriguing virus-host co-evolutionary episode.

### Artiodactyla A3 versus their lentiviruses

To evaluate the antiviral activity of A3 proteins of Artiodactyla such as cattle and sheep, cell-based experiments have been performed using a viral vector system based on HIV-1 and murine leukemia virus and expression plasmids for the A3Z3 and A3Z2Z3 proteins derived from cattle (*Bos taurus*) and sheep (*Ovis aries*) [80–82]. These studies revealed that the A3Z3 and A3Z2Z3 proteins of both cattle and sheep have the ability to suppress the infectivity of *vif*-deleted HIV-1 and murine leukemia virus (a prototype retrovirus in mice) [80–82]. Cattle A3Z3 is degraded by the Vif proteins of BIV (strain BIM127), MVV (strain Iceland), and SIVmac (strain 239) but not by those of HIV-1 (strain LAI) and FIV (strain NCSU) [80, 81]. On the other hand, sheep A3Z3 is degraded by the Vif proteins of MVV (strain Iceland) and HIV-1 (strain LAI) but not by those of SIVmac (strain 239), BIV (strain BIM127) and FIV (strain NCSU) [80, 81]. These findings imply that the Vif-A3Z3 interaction can be promiscuous and that each lentiviral Vif protein is optimized to antagonize the A3Z3 protein of its mammalian host.

#### Bovine A3 versus BIV and JDV

At least 2 lineages of lentivirus have been isolated in bovids: BIV and JDV. BIV was isolated in 1972 in the United States from cattle (*Bos taurus*) displaying persistent lymphocytosis [83]. However, subsequent studies reported that the disorders caused by BIV infection in cattle seem relatively mild compared to those that occur in humans as a result of HIV-1 infection [1, 84, 85]. In contrast, sporadic outbreaks of JDV infection that result in severe disorders and high mortality have occurred in Bali cattle and domesticated banteng (*Bos javanicus*), in certain islands of Indonesia since 1964 [86]. JDV was subsequently identified as the causative agent of severe disorders in Bali cattle in 1993 [87]. Serosurveillance studies reported that BIV infection appears worldwide in cattle [88–94], while JDV infection is endemic in Bali cattle, banteng, and cattle of the islands of Southeast Asia including Indonesia and Malaysia [86, 87, 95, 96].

To address the co-evolutionary dynamics of bovine A3 and the two bovine lentiviruses, BIV and JDV, a recent study determined the sequences of three *A3* genes (i.e., *A3Z1, A3Z2,* and *A3Z3*) of various bovids belonging to the tribe *Bovini*, which includes the genera *Bos* and *Bison*: cattle (*Bos taurus*), zebu (*Bos indicus*), banteng (*Bos javanicus*), gaur (*Bos gaurus*), yak (*Bos grunniens*), European bison (*Bison bonasus*) and American bison (*Bison bison*), as well as the genus *Bubalus* (water buffalo [*Bubalus bubalis*]) [97]. Molecular phylogenetic analyses inferred that bovine *A3Z3* is under strong positive selection and that the amino acids at positions 32, 62, and 92 have been positively selected [97]. Intriguingly, cell-based assays using a *vif*-deleted HIV-1-based reporter system, BIV Vif (strain R27) and JDV Vif (strain Tabanan/87) expression plasmids and respective bovine A3Z3 proteins revealed that gaur A3Z3 is specifically resistant to JDV-mediated degradation [97]. The resistance of gaur A3Z3 to JDV Vif is attributed to 3 codons at position 32, 62, and 92, all of which show evidence of positive selection [97]. As the A3Z3 protein of the MRCA (most recent common ancestor) of cattle, banteng, zebu and gaur, which has been estimated using a molecular phylogenetic method, is sensitive to JDV Vif-mediated degradation [97], the resistance of gaur A3Z3 was found to be acquired after diverging from the other bovids approximately 2.6 million years ago [98]. Investigations on paleontology [99] and molecular phylogenetic [98] have suggested that the tribe *Bovini* originated in Asia. Because JDV is an endemic lentivirus in Southeastern Asia [86, 87, 95, 96], these findings provide the first evidence suggesting that an evolutionary arms race between lentivirus (JDV or its ancestral virus) and mammals (bovids) occurred in Asia in the past.

#### Ovine and caprine A3 versus SRLV

During the 1930s through the 1950s, a pathogenic agent was identified in sheep (*Ovis aries*) in Iceland [100], and subsequent investigations in the early 1970s led to the isolation of MVV as the first lentivirus [101, 102]. CAEV was isolated from goats (*Capra hircus*) [1, 103, 104]. Despite their initial classification as distinct viruses subsequent genetic analyses of MVV and CAEV indicated that these viruses cluster closely [1, 105–107]. Therefore, MVV and CAEV have recently been classified into a single group called SRLV.

Sheep A3Z3 and A3Z2Z3 have the ability to impair *vif*-deleted HIV-1 infection, and these proteins are degraded by MVV Vif (Icelandic strain 1514) [80, 81]. In addition, both the sheep and goat A3Z2Z3 proteins are degraded by the Vif proteins of MVV (strain 1514) and CAEV (strains Cork, 1GA, and Roccaverano) [108]. Both sheep and goats are ruminants that belong to the subfamily *Caprinae*, and current evidence indicates that sheep split from goats approximately 4 million years ago [109]. Therefore, in contrast to felids and bovids, SRLV may have co-evolved with ruminants.

## Vif co-factors

Throughout lentiviral lineages, the *vif* gene serves the same functional purpose of degrading antiviral A3 proteins. However, there is only ~25% conservation of its genetic sequence [81]. Despite the significant divergence of this genetic sequence, all lentiviral Vifs are capable of hijacking the cullin-RING ubiquitin ligase (CRL) complex, which consists of cullin E3 ubiquitin ligase (CUL; CUL2 or CUL5), ring box protein 2 (RBX2) and elongin B/C (ELOB/C) [81, 110, 111]. Although the *vif* sequence is highly divergent, the S/TLQ motif is highly conserved in all lentiviral Vif proteins, and Vif interacts with the CRL complex in an S/TLQ motif-dependent manner [81].

In 2012, two groups identified core binding factor subunit β (CBFB) as the co-factor necessary for PLV Vif to degrade host A3 proteins [110, 111]. PLV Vif, CBFB and ELOB/C form a substrate adaptor for CUL5 and RBX2, which allows the Vif interaction with suitable and susceptible A3 proteins [110]. Vif is the adaptor between the A3 proteins and the CRL complex [111]. CBFB is required for PLV Vif to degrade host A3s but is dispensable for other lentiviral Vif proteins [81, 110–112].

A comparative approach combining proteomic, biochemical, structural, and virological techniques conducted by Kane et al. [81] identified cyclophilin A (CYPA; also known as peptidylprolyl isomerase A) as the co-factor for MVV Vif. The requirement of CYPA is specific for MVV Vif (strain Iceland) [81], and a subsequent study has recently revealed that CAEV (strains Cork, 1GA, and Roccaverano), in addition to MVV Vif (strain 1514), also bind to CYPA [108]. These observations indicate that the CYPA requirement for host A3 degradation is a common feature of SRLV Vif proteins, although PLV Vif does not appear to need CYPA. Moreover, a combination approach of molecular phylogenetic and structural techniques has suggested that mammalian CBFB and CYPA are evolutionarily and structurally conserved [108]. Therefore, lentiviral Vif may have evolved to utilize evolutionarily and structurally stable proteins to degrade host A3 proteins [108]. In contrast to PLV and SRLV, it is intriguing that BIV Vif activity does not appear to be reliant on any co-factors, and no co-factors have yet been identified for FIV Vif activity [81]. These insights further suggest that each lentiviral Vif has evolved an individual strategy to adapt to each host mammal.

## Future perspective: when and how did the *A3* and *vif* genes emerge?

Here, we described the co-evolutionary relationship between mammalian *A3* genes and exogenous lentiviruses. However, many intriguing questions remain: when and how was the *A3* gene acquired in mammals? When and how did lentiviruses acquire the *vif* gene? Was *vif* gene acquired for the purpose of combating host A3s? Or did Vif perform other functions in lentivirus replication that were supplanted? Why is *vif* gene lacking in EIAV?

Regarding the origin of *A3* genes; A3 is known as a component of AID/APOBEC family. AID (activation-induced cytidine deaminase) is a nucleotide mutator contributing to the somatic hypermutation of immunoglobulin genes in B cells, while APOBEC1 edits the mRNA encoding apolipoprotein B that is expressed in small intestine (reviewed in [113, 114]). Since both AID and APOBEC1 are commonly encoded in all mammals as well as birds and reptiles [5], it is plausible to speculate that these genes can be the origin of *A3* genes in mammals. On the other hand, it is known that *A3* genes are not present in opossums (Fig. 2) [115], suggesting that *A3* genes were acquired after (in which family/order of mammals?) divergence with Marsupialia. However, it is unclear whether *A3* gene acquisition occurred in the common ancestor of Eutheria or Boreoeutheria (see Fig. 2). Moreover, we still do not know how many *A3* genes are encoded in the other mammals such as bats and elephants (Fig. 2). Particularly, the mammals belonging to the order Chiroptera (e.g., microbats and megabats) are highly divergent [116]. Therefore, it is plausible to assume that Chiroptera *A3* genes exhibit a high level of diversity. This information will be useful for considering the evolutionary scenario of *A3* acquisition/duplication, which is likely to depend on the evolutionary relationship between lentiviruses and these hosts. Furthermore, Ikeda et al. [117] have recently demonstrated that opossum A1 possesses the activity to impair the replication of lentiviruses and retroelements, suggesting that certain marsupial AID/APOBEC family proteins potently exhibit compensatory activity to limit lentiviral replication instead of A3. Nevertheless, it is still intriguing that the duplications of *AID* and *A1* genes have not been found and that the gene duplication of *AID/APOBEC* family in mammals is specifically occurred in *A3* genes.

The driving force and moment of *A3* duplication are still both unclear. In contrast to the *Z1* and *Z2* domains, it is intriguing that the duplication of *Z3* domain has not been observed in any mammals (Fig. 2). This is reminiscent of the “Kondrashov hypothesis” (also known as “deterministic mutation hypothesis”), which assumes that the majority of deleterious mutations are of small effect and that each subsequent mutation has an increasingly large effect on host fitness [118]. According to this concept, duplication of the *A3Z3* gene may be evolutionarily constrained due to deleterious effects for the host organism. The possibility of host A3Z3 toxicity is further supported by findings related to the *A3H* (the ortholog to *A3Z3* in primates) gene of human and chimpanzee. Human has seven *A3H* haplotypes, but four are not expressed at the protein level [42]. In contrast, chimpanzee *A3H* is monomorphic, and this protein is expressed [42], suggesting that there is a cost to maintain multiple copies of functional A3H. In fact, a recent paper has revealed that human A3H likely contributes to cancer mutagenesis [119]. Therefore, functional A3H has been evolutionarily lost in humans after the divergence from chimpanzees due to its toxicity.

Similar to mammalian *A3* genes, the origin of lentiviral *vif* genes is also unclear. In this regard, endogenous lentiviruses have been detected in the genomes of various mammals, including European rabbits (*Oryctolagus cuniculus*) [120], lemurs belonging to two different genera (*Microcebus* and *Cheirogaleus*) [121, 122], and ferrets (*Mustela putorius furo*) [123, 124]. More intriguingly, all endogenous lentiviruses ever detected appear to encode putative *vif* sequences [121–123]. Considering that all exogenous lentiviruses, with the exception of EIAV in horses, also possess this accessory gene, *vif* gene is an ancient lentiviral component with crucial importance to maintaining lentivirus infections worldwide.
